# P-2342. Dynamics of indirect protection against SARS-CoV-2 infection through vaccine- and infection-acquired immunity

**DOI:** 10.1093/ofid/ofae631.2494

**Published:** 2025-01-29

**Authors:** Sophia T Tan, Isabel Rodríguez-Barraquer, Ada T Kwan, Seth Blumberg, Justine Hutchinson, David Leidner, Joseph A Lewnard, David Sears, Nathan C Lo

**Affiliations:** Stanford University, San Francisco, California; University of California San Francisco, San Francisco, California; University of California San Francisco, San Francisco, California; University of California, San Francisco; California Correctional Health Care Services, Elk Grove, California; California Department of Corrections and Rehabiliation, Sacremento, California; University of California Berkeley, Berkeley, California; University of California, San Francisco; Stanford University, San Francisco, California

## Abstract

**Background:**

Early investigation revealed that COVID-19 vaccines confer indirect protection to fully susceptible and unvaccinated persons, defined as a reduced risk of SARS-CoV-2 infection among social contacts of vaccinated individuals. However, indirect protection from infection-acquired immunity and its comparative strength and durability with vaccine indirect protection in the current epidemiologic context of high levels of vaccination, prior infection, and novel variants are not well characterized, especially in high risk settings such as prisons.

**Table 1.**
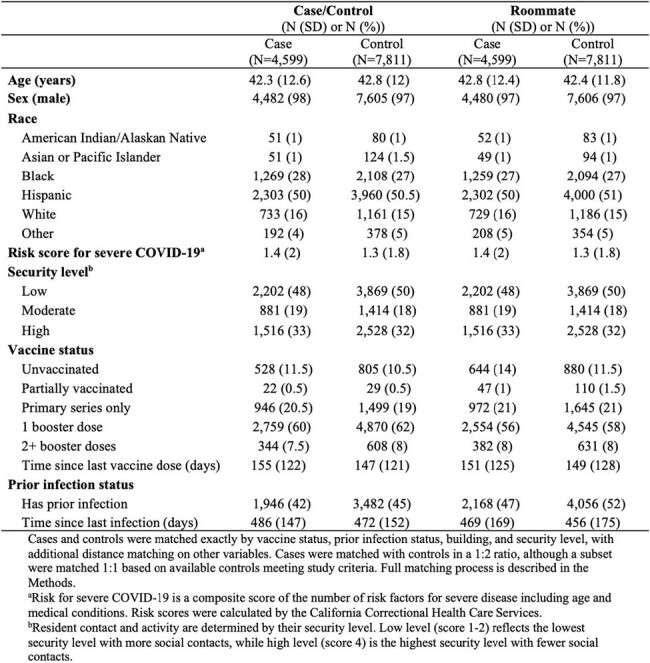
Characteristics of the study population of COVID-19 cases, matched controls, and their roommates in California prisons.

**Methods:**

We conducted a retrospective test-negative case-control study using anonymized data from a surveillance program of 177,319 residents across 35 California state prisons to measure vaccine-derived and infection-acquired indirect protection from March 1, 2020, to December 15, 2022. We defined indirect protection as an individual's risk of infection based on their roommate's vaccine and/or prior infection status. Cases (new SARS-CoV-2 infection) and controls (negative SARS-CoV-2 test) were matched by time (tests within two days), vaccination (by dose and timing), prior infection (including by timing), building, and demographics. We used a conditional logistic regression and estimated indirect protection as one minus the adjusted odds ratio. The study was approved by the Stanford IRB.**Figure 1.** Study population flow chart.

**Figure:**
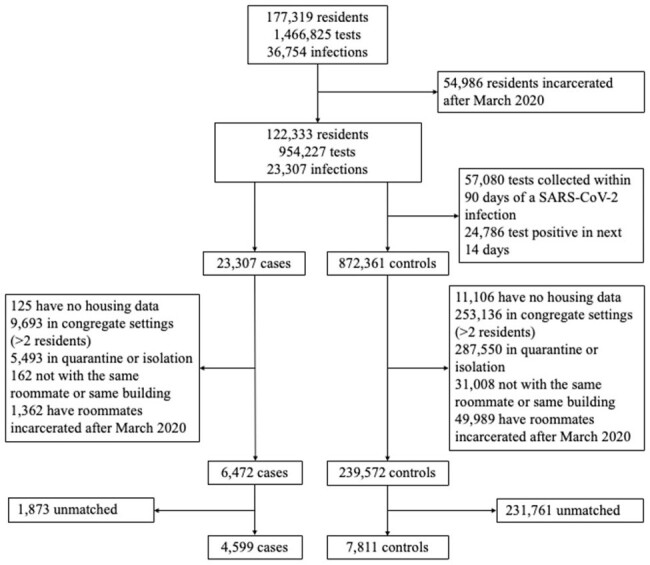

We designed a test-negative case control study to measure the indirect protection provided by COVID-19 vaccination and/or infection-acquired immunity. We analyzed anonymized retrospective data from a SARS-CoV-2 surveillance program of residents incarcerated in the California state prison system from December 15, 2021, to December 15, 2022. We identified individuals with a new SARS-CoV-2 infection (cases) and individuals with a negative SARS-CoV-2 test (controls) during the same time and residing in the same building. Cases and controls were required to co-reside in rooms with a single other resident in the 3-6 days leading up to test collection, to account for the latent period from exposure to detectable infection. We required cases and controls and their roommates to be incarcerated since March 2020 to ensure a complete record of prior infection over the pandemic. Cases and controls were matched in a 1:2 ratio based on multiple characteristics (although a subset was only matched 1:1 based on available controls meeting study criteria), including vaccination and prior infection status. We then evaluated differences in SARS-CoV-2 infection outcome in cases/controls based on the vaccine and prior infection history of their roommate. The sample size of the study population is shown at various stages of applying the study criteria and matching.

**Results:**

We estimate that co-residing with an individual with any vaccine-derived protection or infection-acquired immunity yields 23% (14-32%) and 18% (10-25%) indirect protection against Omicron variant SARS-CoV-2 infection, respectively. Vaccine-derived indirect protection is strongest within three months post-vaccination (30%) and subsequently wanes, whereas infection-acquired immunity provides nearly 40% indirect protection for 6 months after SARS-CoV-2 infection, with measurable indirect protection persisting for over one year. Variant-targeted vaccines (bivalent formulation including Omicron subvariants BA.4/BA.5) confer strong indirect protection for at least three months [51% (18-71%)].**Figure 2.** Overall vaccine-derived and infection-acquired indirect protection to close social contacts against SARS-CoV-2 infection.

**Figure:**
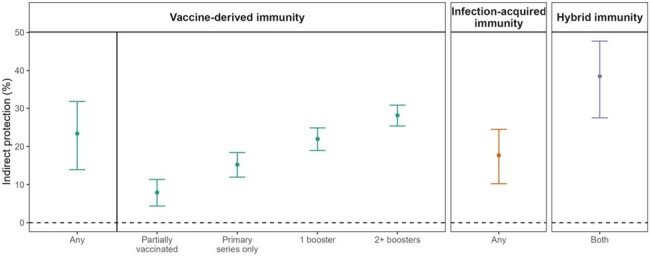

Applying a test-negative case control design, we estimated the indirect protection that COVID-19 vaccination and infection-acquired immunity provided to their close social contact (roommate). We defined indirect protection as change in risk of SARS-CoV-2 infection in an individual based on their roommate’s vaccine and prior infection status. Residents in California state prisons were found to be less likely to be infected by the Omicron SARS-CoV-2 variant if they co-resided with an individual with vaccine-derived and/or infection-acquired protection. The mechanism of protection is likely that individuals with vaccination and/or prior infection are less likely to become infected (and then transmit infection) or are less infectious upon breakthrough infection or reinfection. For residents with hybrid immunity during the study period, most were more likely to be recently vaccinated than recently infected. We plotted the mean (point estimate) and associated 95% confidence intervals (bars) for indirect protection. We defined vaccination both as a binary variable and by dose and infection-acquired immunity as binary. Separate regression models were fit for binary vaccination and binary infection, vaccination by dose, and hybrid immunity.

**Conclusion:**

These results have important implications to understanding transmission dynamics of SARS-CoV-2 and can guide vaccine policy and public health measures, especially in high-risk environments such as prisons.**Figure 3.** Comparative strength and durability of vaccine-derived and infection-acquired indirect protection to close social contacts against SARS-CoV-2 infection.

**Figure:**
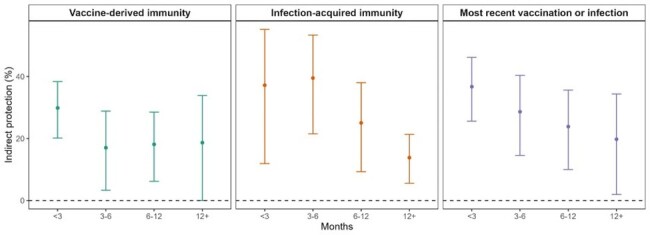

We estimated the strength and durability of indirect protection that COVID-19 vaccination and infection-acquired immunity provided to their close social contact (roommate). Residents in California state prisons were found to have benefit (e.g., less likely to be infected by the Omicron SARS-CoV-2 variant) when residing with an individual with infection-acquired or vaccine-derived protection; both sources of indirect protection waned over time, but infection-acquired protection yielded stronger and more durable indirect protection. We estimated indirect protection based on time since last vaccine dose (left), time since last SARS-CoV-2 infection (middle), and time since either most recent vaccine or infection (right). We plotted the mean (point estimate) and associated 95% confidence intervals (bars) for indirect protection. We fit separate models for vaccine-derived immunity, infection-acquired immunity, and most recent vaccination or infection.

**Disclosures:**

Seth Blumberg, MD PhD, International Responder Systems: Advisor/Consultant Joseph A. Lewnard, PhD, Merck, Sharp, & Dohme: Grant/Research Support|Merck, Sharp, & Dohme: Honoraria|Pfizer: Advisor/Consultant|Pfizer: Grant/Research Support|Pfizer: Honoraria|Pfizer: Speaker fees|Valneva: Honoraria|VaxCyte: Honoraria

